# Efficacy of postoperative adjuvant hepatic artery infusion chemotherapy for hepatocellular carcinoma in microvascular invasion: a propensity-matched score

**DOI:** 10.3389/fsurg.2025.1619772

**Published:** 2025-10-03

**Authors:** Xu Feng, Xinhua Wu, Kai Chen, Yupei Ao, Zhengrong Shi, Yixuan Gong

**Affiliations:** 1 Department of Hepatobiliary Surgery, The First Affiliated Hospital of Chongqing Medical University, Chongqing, China; 2 Health Screening Centre, Chongqing Western Hospital, Chongqing, China; 3 Department of Hepatobiliary Surgery, The Sixth People’s Hospital of Deyang, Deyang, Sichuan, China

**Keywords:** hepatocellular carcinoma, radical liver resection, microvascular invasion, hepatic arterial infusion chemotherapy, propensity score-matching

## Abstract

**Background:**

Patients with hepatocellular carcinoma (HCC) and microvascular invasion (MVI) still have high rates of recurrence and poor survival outcomes after radical resection. This study aims to investigate the effect of postoperative adjuvant hepatic arterial infusion chemotherapy (PA-HAIC) on the recurrence of HCC patients with MVI after radical liver resection (LR).

**Materials and methods:**

This study retrospectively evaluated patients with HCC who underwent LR with MVI at the Hepatobiliary Surgery Department of the First Affiliated Hospital of Chongqing Medical University from 1 January 2020 to 30 June 2024. The recurrence-free survival (RFS) of patients who received PA-HAIC was compared with that of patients who only received LR by propensity score- matching (PSM), and subgroup analyses were performed to compare the efficacy of PA-HAIC for patients in different subgroups based on patient combined risk factors for recurrence, patients' age and the number of PA-HAIC treatments received.

**Results:**

A total of 175 HCC patients with MVI who underwent LR were enrolled in this study, including a total of 72 patients in the PA-HAIC group and 103 patients in the LR group, and after PSM, 67 patients were matched in the PA-HAIC and LR groups, respectively. In the entire cohort, the median RFS (mRFS) were 33.00 months (95% CI, 29.32–36.68 months) and 15.00 months (95% CI, 11.58–18.51 months) for patients in the PA-HAIC and LR groups, respectively (*p* < 0.001). In the PSM cohort, the mRFS was 33.00 months (95% CI, 28.74–37.26 months) and 18.00 months (95% CI, 16.25–19.75 months) for patients in the PA-HAIC and LR groups, respectively (*p* < 0.001). When stratifying patients based on combined risk factors in the entire cohort, in cases where MVI + tumor diameter ≥5 cm (MVID), MVI + multiple tumor (MVIN), and MVI + tumor diameter ≥5 cm + multiple tumor (MVID + N), patients in the PA-HAIC group showed better mRFS than those in the LR group. Within the PA-HAIC group, there was no statistically significant difference in mRFS among patients with MVI alone, MVID, MVIN, and MVID + N. The conclusions of the PSM cohort are consistent. Furthermore, in patients aged ≤55 years, PA-HAIC significantly improved patient mRFS (PA-HAIC group: 32.00 months, 95% CI: 27.61–36.39 months vs. LR group: 13.00 months, 95% CI: 6.48–19.52 months, *p* < 0.001). In addition, patients who received two PA-HAIC treatments had significantly better mRFS compared to those who received only one PA-HAIC treatment (36.00 months, 95% CI 28.26–43.74 months vs. 31.00 months, 95% CI 21.34–40.66 months, *p* = 0.045). Also, the mRFS of patients who received three or more PA-HAIC treatments was similar to that of patients who received two HAIC treatments (*p* = 0.707).

**Conclusions:**

PA-HAIC is beneficial for HCC patients with MVI after radical liver resection, and patients aged ≤55 years with MVI + tumor diameter ≥5 cm, MVI + multiple tumors or MVI + tumor diameter ≥5 cm + multiple tumors should receive at least two PA-HAIC treatments.

## 1 Introduction

Primary liver cancer is the sixth most common cancer globally and the second leading cause of cancer-related deaths, with a particularly high prevalence in resource-limited developing countries. Hepatocellular carcinoma (HCC) accounts for approximately 75%–85% of all primary liver cancer cases (1, 2). In China, HCC is the fifth most common cancer and the second leading cause of cancer-related deaths (3). Chronic Hepatitis B Virus (HBV) or Hepatitis C Virus infection, alcohol consumption, exposure to aflatoxins, and non-alcoholic fatty liver disease are common risk factors (4–7). For HCC treatment, radical methods such as ablation, radical liver resection (LR), and liver transplantation are the primary choices (8–10). However, due to the inherent heterogeneity of HCC, there still remains a high recurrence rate even after LR. According to statistics, the recurrence rate within 5 years after radical resection remains as high as 50%–70%. Compared to patients without recurrence, the 5-year survival rate of patients with HCC recurrence is reduced by approximately 24% (11–13). Recurrence of HCC within two years post-surgery is referred to as early recurrence (Type 1), primarily due to residual microscopic lesions after surgery. Recurrence after two years is considered late recurrence (Type 2), originating from new carcinogenesis within the liver tissue (13, 14). Early recurrence has a significant impact on patient prognosis. Therefore, effectively preventing early postoperative recurrence of HCC has become crucial in improving the prognosis of HCC patients.

It is widely accepted that certain factors increase the risk of early recurrence of HCC after LR. These factors include multiple tumor or satellite foci, tumor diameter ≥5 cm, poor tumor differentiation, microvascular invasion (MVI), and macrovascular invasion (15–17). Several studies have demonstrated that postoperative adjuvant hepatic arterial infusion chemotherapy (PA-HAIC) is greatly effective in patients undergoing radical resection, which significantly reduces the recurrence rates and prolongs the overall survival time (OS) of patients (18–21). The diagnosis of MVI is mainly based on postoperative pathological confirmation, and the diagnostic criterion is the microscopic sighting of clusters of cancer cell nests in the lumen of endothelium-lined blood vessels, most commonly found in small branches of the portal vein or blood vessels within the tumor membrane within the paracancerous liver tissue (22, 23). There is not much literature comparing the efficacy of PA-HAIC in patients with MVI, therefore this study aims to investigate the efficacy of PA-HAIC on the recurrence of HCC patients with MVI after LR.

## 2 Materials and methods

### 2.1 Patients

This study retrospectively evaluated patients with HCC who underwent LR with MVI at the Hepatobiliary Surgery Department of the First Affiliated Hospital of Chongqing Medical University from 1 January 2020 to 30 June 2024. The study was conducted in accordance with the Declaration of Helsinki, and was approved by the institutional ethics committees of our medical center (K2014-039-01). The study was retrospective and no further patient consent was required.

Patients who met the following criteria were enrolled: (1) HCC stage BCLC 0-B prior to surgery; (2) postoperative pathology confirmed hepatocellular carcinoma with MVI; (3) radical liver resection (negative margins confirmed by pathology); (4) without any preoperative anticancer treatments; (5) HAIC treatment only or no treatment after surgery; (6) no history of other malignancies or autoimmune diseases. Exclusion criteria: (1) R1 resection (postoperative pathology suggesting positive margins) or preoperative imaging suggesting extrahepatic metastases; (2) non-HCC confirmed by postoperative pathology; (3) with MVI negative or macrovascular invasion; (4) Preoperative anti-tumor therapies; (5) Post-operative treatments other than HAIC or HAIC in combination with other anti-tumor therapies; (6) Patients who relapsed or died within 60 days of surgery.

### 2.2 Radical liver resection and PA-HAIC

All patients underwent routine preoperative examinations including ultrasound, enhanced electronic CT or enhanced magnetic resonance imaging (MRI) to assess tumor diameter, BCLC stage, resectable extent and residual liver volume. In addition, liver function was assessed using the Child-Pugh classification and cirrhosis using ICG15 in all patients. The hepatectomy method contains non-anatomical resection and anatomical resection, and the surgical technique used depends on the location and distribution of the tumor. Anatomical hepatectomy is the complete resection of the segment of liver with the tumor or the segment of liver limited by the branches of the portal vein of the tumor. Non-anatomical hepatectomy is the resection of the tumor and part of the non-tumor liver parenchyma (24, 25). Radical liver resection was defined as the complete removal of all detected tumors without involving any major branch of the portal or hepatic veins, without invasion of adjacent organs and without lymph node or distant metastasis, and tumor-free margins confirmed by histopathology (10). Postoperative adjuvant modalities, including HAIC, are recommended for all patients with MVI. However, patients will decide whether to receive one or more adjuvant treatments, and the number of treatments, based on their medical compliance, economic status or other social factors. Before receiving adjuvant therapy, patients and their families must be fully informed about the relevant treatment modalities and adverse effects, and sign informed consent forms. Meanwhile, all patients with hepatitis B virus need to be treated with antiviral drugs.

PA-HAIC: Patients were re-evaluated approximately 4–6 weeks post-operatively for blood counts, liver and kidney function, alpha-fetoprotein, CT or MRI enhancement of the epigastric region, and were treated with PA-HAIC after assessment of no tumor recurrence and absence of obvious contraindications. In the Seldinger technique, a hepatic artery catheter is placed through the femoral artery into the appropriate hepatic artery and any suspicious tumor staining in the remaining liver is detected by digital subtraction angiography (DSA) or CT angiography. The catheter was left in place and connected to the infusion pump on the ward. The following chemotherapy drugs are pumped continuously: oxaliplatin 85 mg/m^2^ from 0 to 3 h on day 1, leucovorin 400 mg/m^2^ from 3 to 4.5 h on day 1, 5-fluorouracil 400 mg/m^2^ from 4.5 to 6.5 h on day 1, and 5-fluorouracil 2,400 mg/m^2^ over 46 h from days 1 to 3 (26). The patient had almost complete restriction of movement of the right lower limb during the infusion. At the end of the chemotherapy, the catheter was removed and the puncture site bandaged for 8 h to allow movement of the right lower limb, then the blood and liver functions were re-checked and the patient was discharged from hospital if there were no obvious abnormalities. The interval between two cycles of PA-HAIC was set at 4–5 weeks. If patients were unable to tolerate the treatment due to physical frailty, pain, fever, nausea, vomiting, or myelosuppression, the interval could be appropriately extended and/or the drug dosage adjusted. However, the interval was not allowed to exceed 2 months, and the dosage could not be reduced to less than 75% of the standard dose.

### 2.3 Follow up and outcomes

All patients were followed during outpatient visits or hospitalizations. Follow-up was conducted every 1–2 months during the first 6 months and every 3–6 months thereafter. Approximately two months after LR, at least two imaging modalities-ultrasound, CT, or MRI-were performed to evaluate tumor recurrence. Patients with confirmed recurrence at this time point were excluded from further analysis, while those without recurrence continued regular follow-up. During subsequent follow-up, patients underwent routine blood tests, liver function tests, AFP, and abdominal ultrasound. If recurrence was suspected, contrast-enhanced CT or MRI was performed for confirmation. Recurrence was defined as any tumor nodule confirmed by at least two imaging modalities or by histopathological biopsy. The study endpoint was recurrence-free survival (RFS), defined as the time from LR to the diagnosis of tumor recurrence. All patients were followed until 31 January 2025 or until loss to follow-up or death.

### 2.4 Propensity score-matching

Propensity score-matching (PSM) analysis was involved to minimize alternative factors and sampling bias between the two groups. A 1:1 nearest neighbor matching algorithm was used with a caliper width of 0.02. PSM was performed using SPSS 27.0 statistical software (IBM Corp., Armonk, NY, USA).

### 2.5 Statistical analysis

Statistical analyses were performed with SPSS 27.0. The Shapiro–Wilk test was used to test the normality of continuous variables, and the independent samples *t*-test was used to detect continuous data that followed the normal distribution, expressed as the mean ± standard deviation. The Mann–Whitney *U*-test was used to detect continuous data that were not normally distributed, expressed as median (interquartile range, IQR). Categorical data were detected using the chi-squared test, and expressed as numbers (*n*) and proportions (%). Univariate and multivariate analyses were performed in Cox risk models to identify independent prognostic factors for RFS. Survival analyses were performed using the Kaplan–Meier method, and differences in the survival curves were analyzed using the log-rank test. K-M curves were plotted using R software (version 4.2.1 http://www.r-project.org). *P*-value <0.05 was considered statistically significant.

## 3 Result

### 3.1 Baseline patient characteristics

**Figure 1:**
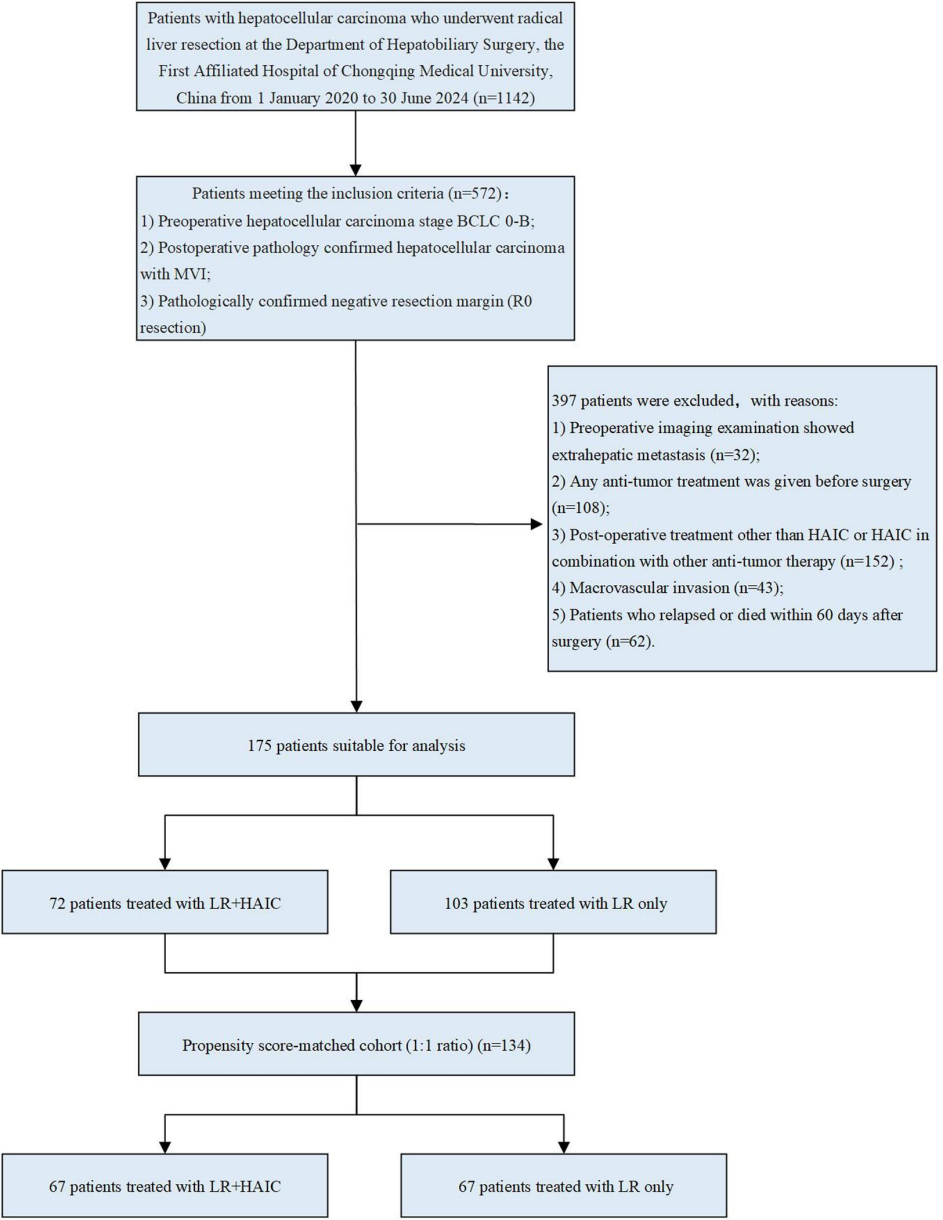
Flowchart of patient selection. HCC, hepatocellular carcinoma; LR, liver resection; HAIC, hepatic arterial infusion chemotherapy; PSM, propensity score- matching.

A total of 175 patients who met the criteria were included in this study, and the flowchart of patient selection is presented in Figure 1.

The entire patient age range was 56.67 ± 11.27 years, with 156 (88.14%) males and 21 (11.86%) females; 72 patients were treated postoperatively with HAIC, with the majority receiving 1–2 HAIC treatments (83.33%), and 103 patients received no postoperative treatment. In the entire cohort, patients in the PA-HAIC group were younger than those in the LR group (53.58 ± 10.78 years vs. 58.96 ± 11.18 years, *p* = 0.002), and no differences were seen in other baseline characteristics. After PSM, 67 patients were matched in the PA-HAIC and LR groups, respectively. The age range of all patients was 54.87 ± 10.06 years, with a total of 118 (88.06%) males and 16 (11.94%) females, and most patients in the PA-HAIC group received 1–2 HAIC treatments (82.09%). The baseline characteristics of the patients in the two groups did not differ significantly. A total of 138 cycles of PA-HAIC were administered to 72 patients. No treatment-related deaths or severe adverse events were observed. All adverse events were mild and alleviated with symptomatic treatment. Baseline information for the entire cohort and PSM cohort patients is summarized in Table 1.

**Table 1:** Baseline characteristics of HCC patients with MVI in different treatment groups.

Characteristics		The entire cohort			The PSM cohort		
		PA-HAIC (*n* = 72)	LR (*n* = 103)	*p*	PA-HAIC (*n* = 67)	LR (*n* = 67)	*p*
Age, years		53.78 ± 10.81	58.88 ± 11.14	0.003	54.85 ± 10.06	54.90 ± 10.14	0.980
Sex male, *n* (%)		62 (86.11)	92 (89.32)	0.520	59 (88.06)	59 (88.06)	1.000
HBsAg-positive, *n* (%)		65 (90.28)	87 (84.47)	0.263	60 (89.55)	60 (89.55)	1.000
Liver cirrhosis yes, *n* (%)		43 (59.72)	59 (57.28)	0.747	40 (59.70)	38 (56.72)	0.726
AFP, ng/ml		52.07 (5.27, 1,134.85)	29.80 (4.20, 332.00)	0.391	29.60 (5.12, 1,197.00)	29.80 (4.60, 300.00)	0.371
Tumor diameter, cm		4.15 (2.70, 6.65)	4.30 (3.10, 7.10)	0.321	4.10 (2.70, 6.70)	4.00 (3.00, 5.80)	0.674
Tumor number, *n* (%)	Single	46 (63.87)	68 (66.02)	0.771	42 (62.69)	51 (76.12)	0.092
	Multiple	26 (36.11)	35 (33.98)		25 (37.31)	16 (23.88)	
Differentiation, *n* (%)	Low	5 (6.94)	10 (9.71)	0.574	3 (4.48)	4 (5.97)	0.839
	Median	56 (77.78)	82 (79.61)		53 (79.10)	54 (80.60)	
	High	11 (15.28)	11 (10.68)		11 (16.42)	9 (13.43)	
BCLC grade, *n* (%)	0 + A1	46 (63.89)	68 (66.02)	0.163	42 (62.69)	51 (76.12)	0.151
	A2	10 (13.89)	6 (5.82)		10 (14.92)	4 (5.97)	
	B	16 (22.22)	29 (28.16)		15 (22.39)	12 (17.91)	
Child-Pugh grade, *n* (%)	A	69 (95.83)	96 (92.20)	0.461	64 (95.52)	63 (94.03)	0.698
	B	3 (4.17)	7 (6.80)		3 (4.48)	4 (5.97)	
Hemoglobin, g/L		142.50 (126.75, 153.50)	138.50 (128.75, 153.00)	0.542	143.00 (126.50, 154.00)	141.00 (130.00, 153.00)	0.841
NLR		2.14 (1.73, 3.23)	2.50 (1.69, 3.85)	0.531	2.42 (1.64, 3.40)	2.49 (1.61, 3.85)	0.991
PLR		101.41 (72.84, 149.82)	112.65 (81.96, 149.92)	0.325	101.40 (72.57, 150.21)	97.70 (74.19, 131.38)	0.690
SII		334.68 (213.73, 495.19)	357.03 (226.49, 613.51)	0.395	323.97 (201.82, 496.50)	295.17 (192.14, 546.48)	0.886
Total protein, g/L		69.42 ± 7.35	68.86 ± 7.37	0.964	69.52 ± 7.55	68.45 ± 6.88	0.401
Albumin, g/L		40.60 (38.75, 44.00)	43.00 (38.00, 45.00)	0.119	40.20 (38.50, 43.50)	43.00 (38.00, 45.00)	0.196
Total bilirubin, umol/L		11.25 (8.85, 16.40)	12.40 (9.38, 16.93)	0.299	11.00 (8.80, 16.40)	13.50 (9.30, 19.90)	0.281
ALT, U/L		34.50 (26.00, 56.75)	34.50 (26.00, 51.00)	0.921	34.00 (24.50, 55.00)	36.00 (27.00, 59.00)	0.310
AST, U/L		34.00 (24.75, 55.25)	34.50 (26.00, 51.00)	0.684	35.00 (26.00, 53.50)	33.00 (27.00, 45.00)	0.949
PT, s		13.80 (13.30, 14.50)	13.75 (13.30, 14.23)	0.891	13.80 (13.30, 14.50)	13.90 (13.30, 14.50)	0.335
Hemorrhage, ml		275.00 (145.00, 500.00)	300.00 (200.00, 500.00)	0.545	300.00 (150.00, 500.00)	300.00 (200.00, 500.00)	0.913
Operating time, minutes		252.00 (203.75, 310.00)	264.50 (213.75, 330.25)	0.226	270.00 (202.50, 312.50)	260.00 (215.00, 310.00)	0.704
Blood transfusion, *n* (%)		7 (9.72)	11 (10.68)	0.837	7 (10.45)	8 (11.94)	0.784
Resection pattern, *n* (%)	Anatomic	48 (66.67)	69 (66.99)	0.964	46 (68.66)	42 (62.69)	0.467
	Nonanatomic	24 (32.33)	34 (33.01)		21 (31.34)	25 (37.31)	
Resection margin ≥1 cm, *n* (%)		72 (100.00)	103 (100.00)	1.000	67 (100.00)	67 (100.00)	1.000
The number of HAIC, *n* (%)	1	22 (30.55)	–	–	19 (28.36)	–	–
	2	38 (52.78)	–		36 (53.73)	–	
	≥3	12 (16.67)	–		9 (17.91)	–	

PSM, propensity score- matching; LR, liver resection; PA-HAIC, postoperative adjuvant hepatic arterial infusion chemotherapy; HBV, hepatitis B virus; AFP, alpha-fetoprotein; ALT, alanine aminotransferase; AST, aspartate aminotransferase; PT, prothrombin time; BCLC, Barcelona clinic liver cancer; NLR, neutrophil/lymphocyte; PLR, platelet/lymphocyte; SII; neutrophil*platelet/lymphocyte; SIRI, neutrophil*monocyte/lymphocyte.

### 3.2 Efficacy analysis

**Figure 2:**
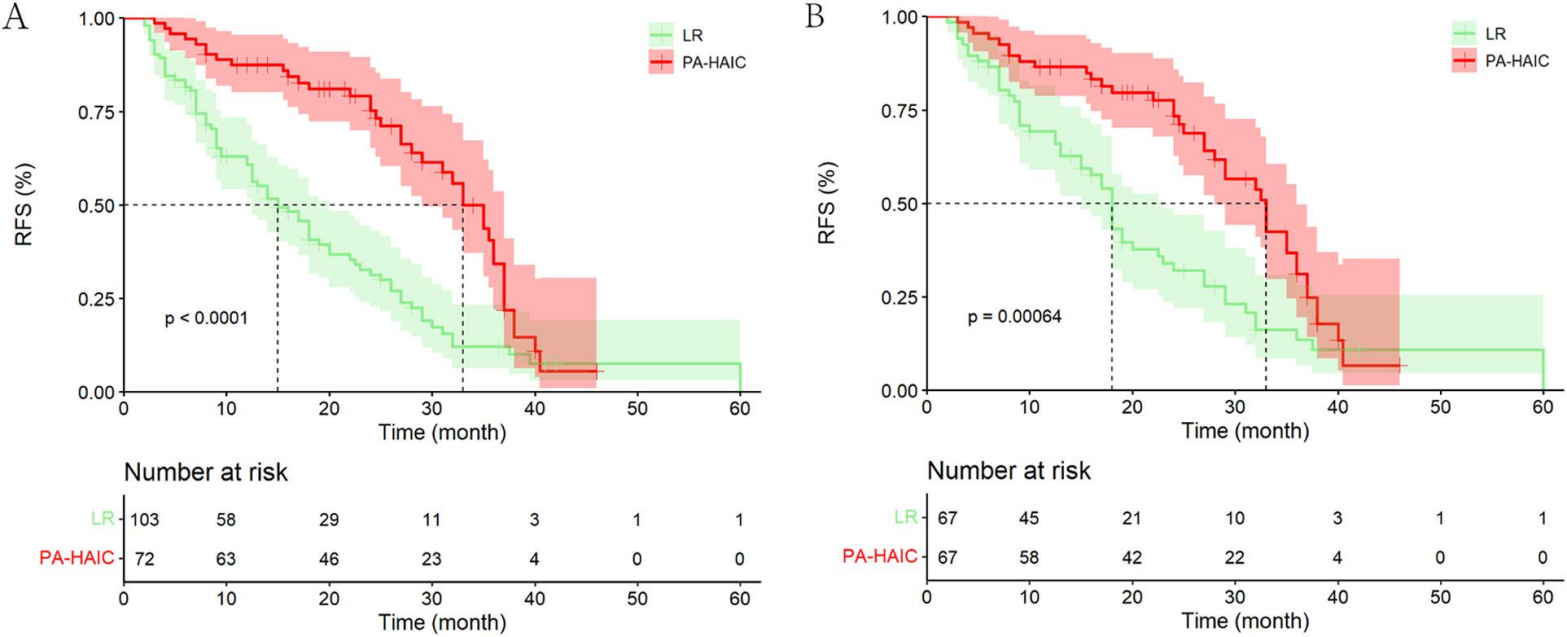
Kaplan–Meier analysis recurrence-free survival of HCC patients with MVI after liver resection. **(A)** The entire cohort, **(B)** the PSM cohort.

The median follow-up time for all patients was 34.00 months (95% CI, 25.98–42.03 months). In the entire cohort, a total of 117 (66.86%) patients relapsed, including a total of 39 (54.67%) patients in the PA-HAIC group and 78 (75.73%) patients in the LR group. The median RFS (mRFS) was 24.00 months (95% CI, 19.90–28.11 months) for all patients, and 33.00 months (95% CI, 29.32–36.68 months) and 15.00 months (95% CI, 11.58–18.51 months) for patients in the PA-HAIC and LR groups, respectively (*p* < 0.001). The 1-, 2- and 3-year RFS rates were 87.50% (95% CI, 79.86%–95.14%), 75.20% (95% CI, 64.42%–85.98%) and 34.30% (95% CI, 19.01%–49.59%) in the PA-HAIC group and 60.80% (51.20%–70.40%), 31.30% (21.30%–41.30%) and 10.10% (2.65%–17.55%) in the LR group, respectively, with statistically significant differences (*p* < 0.001) (Figure 2A).

In PSM cohort, a total of 89 (66.42%) patients relapsed, including a total of 38 (56.72%) patients in the PA-HAIC group and 51 (76.12%) patients in the LR group. The mRFS was 27.00 months (95% CI, 22.70–31.30 months) for all patients, and 33.00 months (95% CI, 28.74–37.26 months) and 18.00 months (95% CI, 16.25–19.75 months) for patients in the PA-HAIC and LR groups, respectively (*p* < 0.001). The 1-, 2- and 3-year RFS rates were 86.50% (95% CI, 78.27%–94.73%), 73.30% (95% CI, 65.07%–81.53%) and 31.10% (95% CI, 22.87%–39.33%) in the PA-HAIC group and 66.10% (57.87%–74.33%), 32.10% (19.95%–44.25%) and 13.50% (3.50%–23.50%) in the LR group, respectively, with statistically significant differences (*p* < 0.001) (Figure 2B).

### 3.3 COX regression analysis

Univariate and multivariate Cox regression analyses were performed in both the entire cohort and the PSM cohort in Table 2 and Supplementary Table S1. In the entire cohort, multiple tumor (2.236, 95% CI 1.487, 3.363), and tumor diameter (1.095, 95% CI 1.020, 1.175) were independent risk factors for RFS. In contrast, compared with LR, PA-HAIC (0.369, 95% CI 0.246, 0.553) were identified as independent protective factors for RFS. In the PSM cohort, it was found that age (0.977, 95% CI 0.953, 0.996), multiple tumor (3.002, 95% CI 1.729, 5.211), tumor diameter (1.162, 95% CI 1.055, 1.256), low differentiation (4.064, 95% CI 1.358, 12.164) were the independent risk factors for RFS. PA-HAIC (0.276, 95% CI 0.165, 0.463) were identified as independent protective factors for RFS.

**Table 2:** Multivariate analysis of RFS in the entire cohort and the PSM cohort.

Characteristics		The entire cohort		The PSM cohort	
		HR (95% CI)	*p*	HR (95% CI)	*p*
Treatment	LR	Reference		Reference	
	PA-HAIC	0.369 (0.246, 0.553)	<0.001	0.276 (0.165, 0.463)	<0.001
Tumor number	Single	Reference		Reference	
	Multiple	2.236 (1.487, 3.363)	<0.001	3.002 (1.729, 5.211)	<0.001
Tumor diameter		1.095 (1.020, 1.175)	0.012	1.162 (1.060, 1.274)	0.001
NLR		1.097 (0.949, 1.268)	0.211	1.094 (0.986, 1.215)	0.091
SII		1.000 (0.999, 1.001)	0.829	–	–
Age		–	–	0.977 (0.953, 0.996)	0.023
Differentiation	High		–	Reference	
	Median	–	–	1.635 (0.766, 3.492)	0.204
	Low	–	–	4.064 (1.358, 12.164)	0.012
PT		–	–	1.291 (0.999, 1.668)	0.051

PSM, propensity score- matching; LR, liver resection; PA-HAIC, postoperative adjuvant hepatic arterial infusion chemotherapy; NLR, neutrophil/lymphocyte; SII, neutrophil*platelet/lymphocyte; PT, prothrombin time.

### 3.4 Stratification of the risk factors

Based on the patients' risk factors for recurrence, patients were classified into combined MVI alone (MVI), MVI + tumor diameter ≥5 cm (MVID), MVI + multiple tumor (MVIN), MVI + tumor diameter ≥5 cm + multiple tumor (MVID + N), and MVI + poor differentiation. Given that the number of patients with MVI + poor differentiation was small in the entire cohort and PSM cohort (5 patients in the PA-HAIC group and 10 in the LR group in the entire cohort, and 3 patients in the PA-HAIC group and 4 patients in the LR group in the PSM cohort), the analysis may be somewhat biased and was therefore not analyzed.

**Figure 3:**
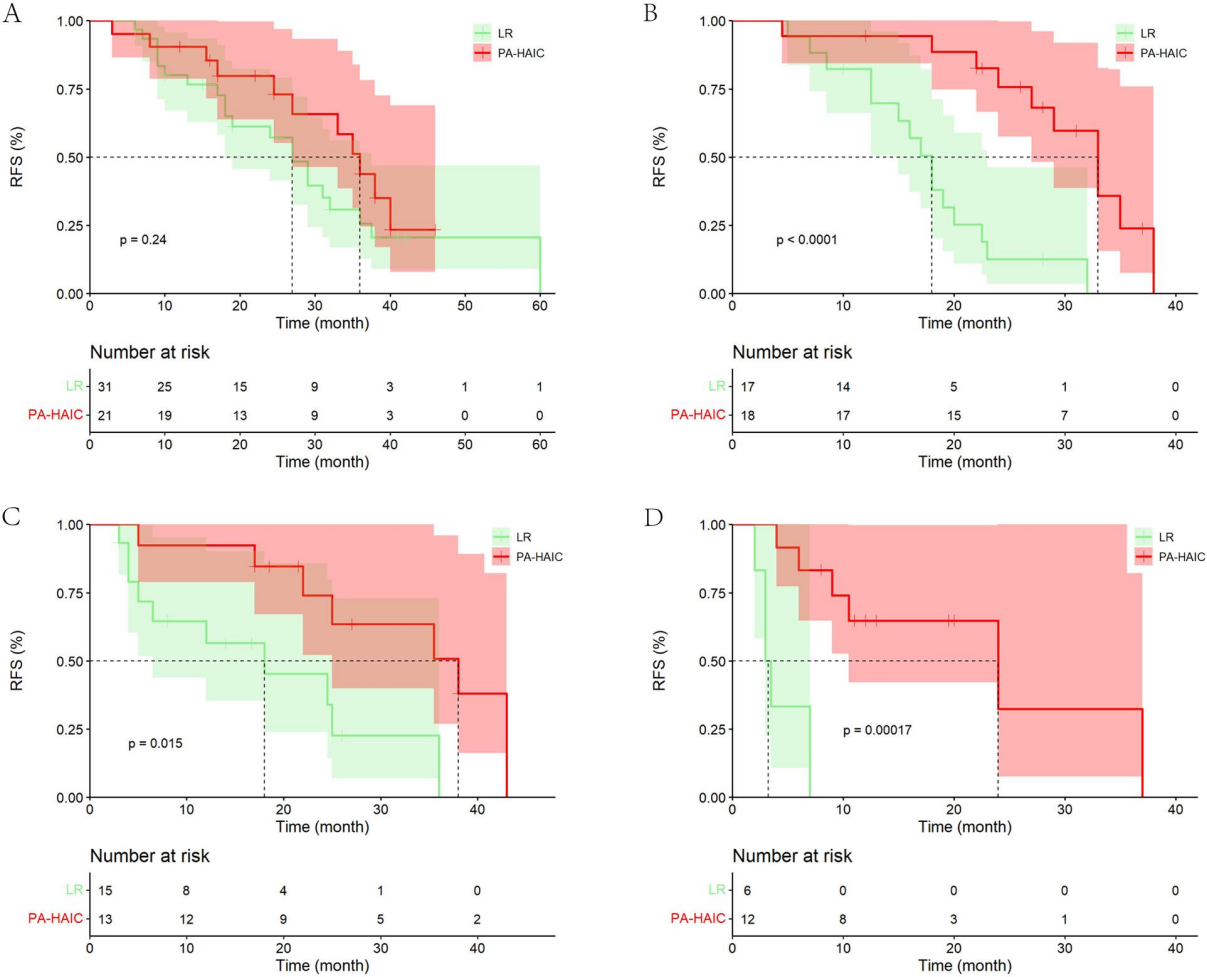
Kaplan–Meier analysis of recurrence-free survival in HCC patients with different risks of recurrence (the PSM cohort). **(A)** With MVI alone, **(B)** with MVI + tumor diameter ≥5 cm, **(C)** with MVI + multiple tumor, **(D)** with MVI + tumor diameter ≥5 cm + multiple tumor.

In the entire cohort, although patients with MVI in the PA-HAIC group had a longer mRFS than those in the LR group, there was no statistical difference (PA-HAIC group: 33.00 months, 95% CI 28.67–37.33 months vs. LR group: 27.00 months, 95% CI 22.66–31.34 months, *p* = 0.200). Considering that the number of patients who relapsed as a percentage of the total subgroup at the follow-up endpoint was lower in the PA-HAIC group than in the LR group (50.00% vs. 67.74%), this finding was required further investigation. For MVID, MVIN and MVID + N, the mRFS of patients in the PA-HAIC group were significantly better than those of the LR group (MVID: 36.00 months, 95% CI 32.51–39.49 months vs. 15.00 months, 95% CI 9.12–20.88 months; MVIN: 38.00 months, 95% CI 21.39–54.61 months vs. 18.00 months, 95% CI 1.99–34.01 months; MVID + N: 24.00 months, 95% CI 5.78–42.22 months vs. 4.00 months, 95% CI 0–8.85 months, respectively). In the PSM cohort, there was a consistent conclusion to be drawn. All of the above data are presented in Figure 3 and Supplementary Figure S1.

**Figure 4:**
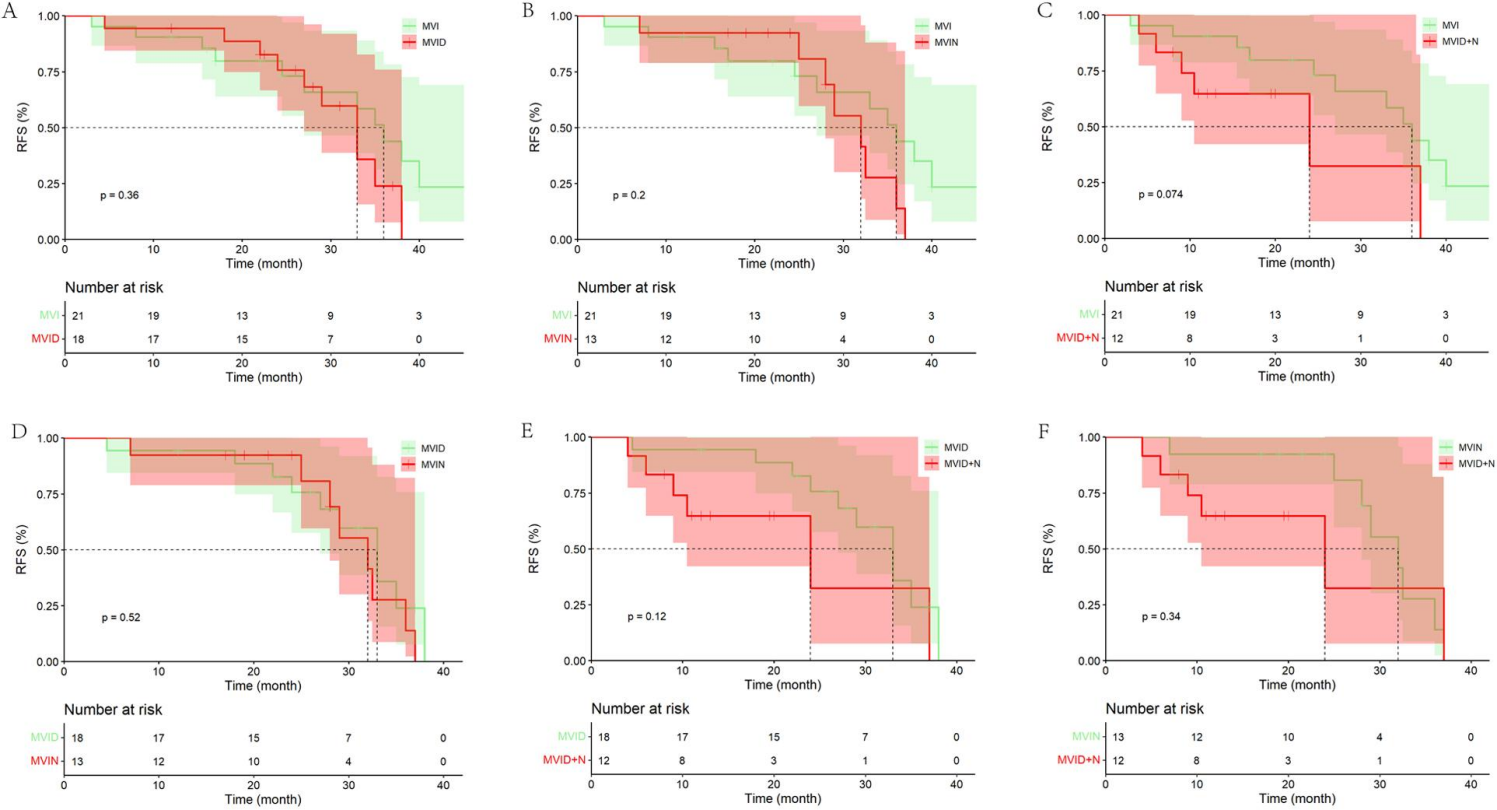
Kaplan–Meier analysis of recurrence-free survival in HCC patients with different risk factors in the PA-HAIC group (the PSM cohort). **(A)** With MVI alone, **(B)** with MVI + tumor diameter ≥5 cm, **(C)** with MVI + multiple tumor, **(D)** with MVI + tumor diameter ≥5 cm + multiple tumor.

It was also found that in the LR groups, the mRFS of patients with MVI, MVI + D/MVI + N and MVID + N progressively decreased and were statistically different. However, no statistical difference was found between patients with MVID and patients with MVIN (Supplementary Table S2). In the PA-HAIC group, there were no statistical differences in mRFS between patients with MVI, MVID, MVIN, and MVID + N (the entire cohort: 33.00 months, 95% CI 28.67–37.33 months; 36.00 months, 95% CI 32.51–39.49 months; 35.00 months, 95% CI 19.97–50.03 months; 24.00 months, 5.78–42.22 months; the PSM cohort: 36.00 months, 95% CI 30.79–41.21 months; 33.00 months, 95% CI 27.98–38.02 months; 32.00 months, 95% CI 24.49–39.51 months; 16.00 months, 95% CI 3.20–28.80 months, respectively), as shown in Figure 4 and Supplementary Figure S2.

### 3.5 Impact of age stratification on patient prognosis

**Figure 5:**
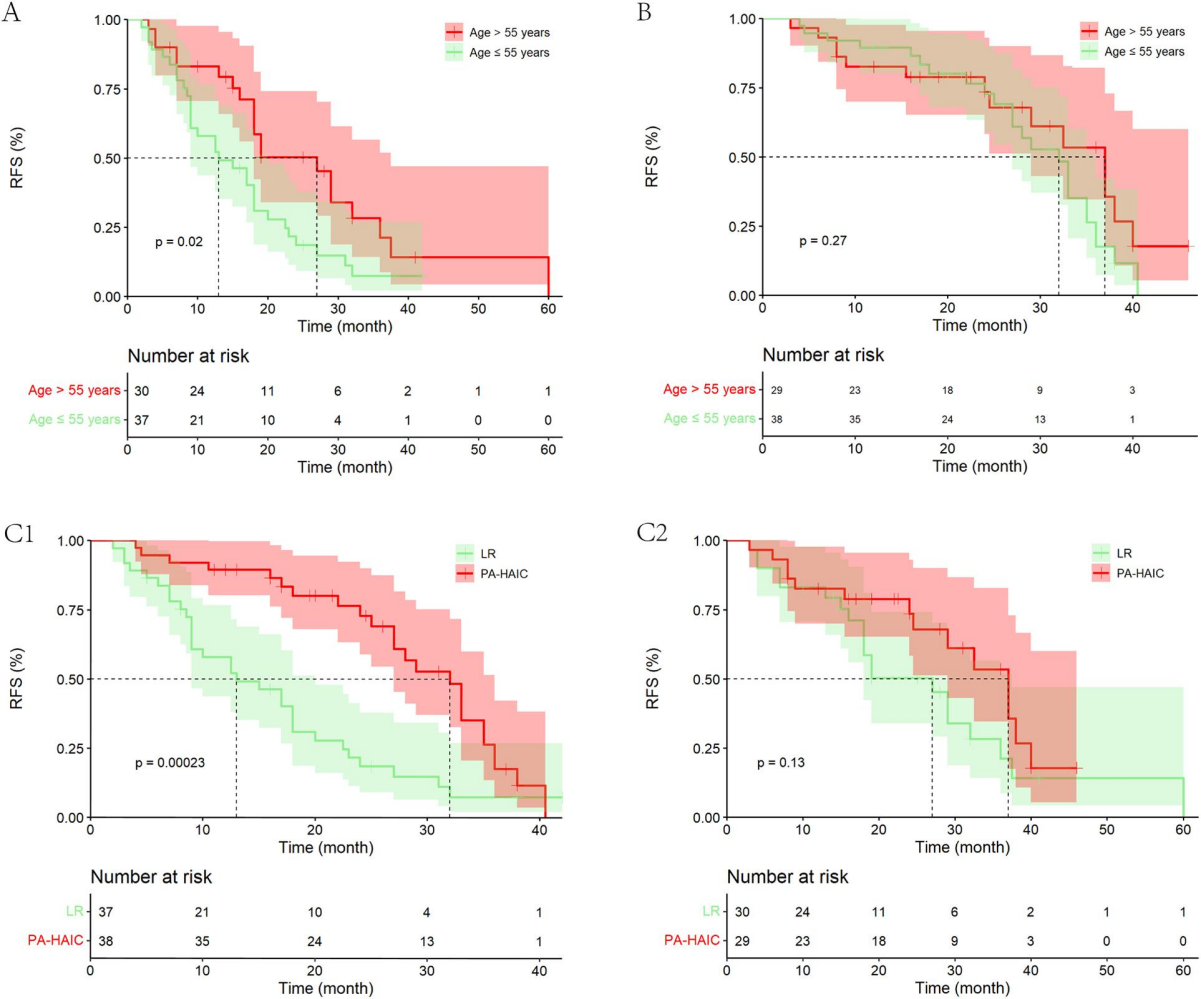
Kaplan–Meier analysis of recurrence-free survival in HCC patients in different age groups. **(A)** Patients age ≤55 years and age >55 years in the LR group, **(B)** patients age ≤55 years and age >55 years in the PA-HAIC group, **(C)** patients in the LR and PA-HAIC groups (1- aged ≤55 years and 2- aged >55 years).

Patients were divided into ≤55 and >55 years groups based on the median age of the PSM cohort. The study found significant difference in mRFS between patients aged ≤55 and >55 years in the LR group (13.00 months, 95% CI 6.48–19.52 months vs. 27.00 months, 95% CI 18.12–35.88 months, *p* = 0.020). In patients aged ≤55 years, PA-HAIC significantly improved patient mRFS (PA-HAIC group: 32.00 months, 95% CI: 27.61–36.39 months vs. LR group: 13.00 months, 95% CI: 6.48–19.52 months, *p* < 0.001), while in patients aged >55 years, mRFS in the PA-HAIC group was better than in the LR group, but not statistically different (37.00 months, 95% CI: 29.00–45.00 months vs. 27.00 months, 95% CI: 18.12–35.88 months, *p* = 0.126). However, the results may have been influenced by the lower recurrence rate of patients in the PA-HAIC group compared to the LR group (48.30% vs. 66.70%). In addition, there was no significant difference in mRFS between patients aged ≤55 and >55 years in the PA-HAIC group (32.00 months, 95% CI: 27.61–36.39 months vs. 37.00 months, 95% CI: 29.00–45.01 months, *p* = 0.130) (Figure 5).

### 3.6 Efficacy of the number of PA-HAIC treatments

**Figure 6:**
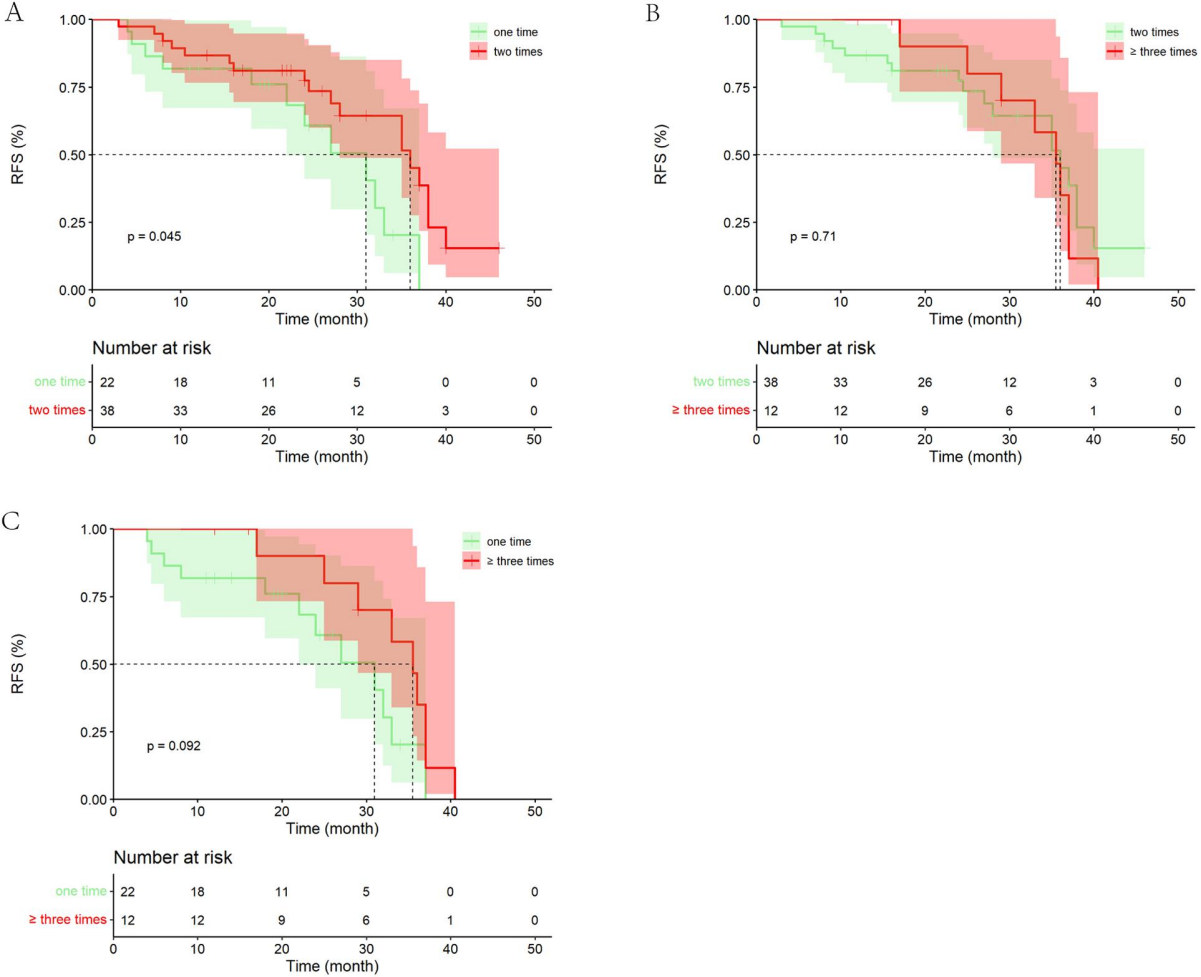
Kaplan–Meier analysis of recurrence-free survival in patients with different number of PA-HAIC. **(A)** One PA-HAIC treatment vs. two PA-HAIC treatments, **(B)** two PA-HAIC treatments vs. ≥three PA-HAIC treatments, **(C)** one PA-HAIC treatment vs. ≥three PA-HAIC treatments.

Among the 72 patients who underwent PA-HAIC treatment, 22 patients received one treatment, 38 patients received two treatments, and 12 patients received three or more treatments. The study discovered that patients who received two PA-HAIC treatments had significantly better mRFS than those who received only one PA-HAIC treatment (36.00 months, 95% CI 28.26–43.74 months vs. 31.00 months, 95% CI 21.34–40.66 months, *P* = 0.045). Although superior to those who received one treatment, the mRFS of patients who received three or more treatments did not show significant difference (35.50 months, 95% CI 31.22–39.68 months vs. 31.00 months, 95% CI 21.34–40.66 months, *p* = 0.092). Furthermore, patients who underwent three or more treatments exhibited a similar RFS to those who received two treatments (mRFS: 35.50 months, 95% CI 31.22–39.68 months vs. 36.00 months, 95% CI 28.26–43.74 months, *p* = 0.707). It is important to note that the limited number of patients (only 12 patients) who received three or more treatments may have influenced the results to some degree (Figure 6).

### 3.7 Treatment after recurrence

By the end of follow-up, 37 patients in the PA-HAIC group had relapsed and 1 patient had died before relapse; in the LR group, 78 patients had relapsed and no patient had died before relapse. Three patients died after relapse in the PA-HAIC group and five patients died after relapse in the LR group. Patients' treatment patterns after relapse are shown in the Table 3.

**Table 3:** Patients’ treatment patterns after relapse.

Treatment patterns	PA-HAIC (*n* = 37)	LR (*n* = 78)
Surgical resection, *n* (%)	4 (10.81)	14 (17.95)
Radiofrequency ablation, *n* (%)	3 (8.11)	4 (5.13)
Targeted therapy, *n* (%)	4 (10.81)	6 (7.69)
TACE/HAIC + Immunotherapy, *n* (%)	6 (16.22)	13 (16.67)
Targeted therapy + Immunotherapy, *n* (%)	10 (27.03)	17 (21.79)
TACE/HAIC + Targeted therapy + Immunotherapy, *n* (%)	10 (27.03)	24 (30.77)

LR, liver resection; PA-HAIC, postoperative adjuvant hepatic arterial infusion chemotherapy.

## 4 Discussion

The development and progression of HCC is a multi-stage, chronic process, with chronic liver disease due to persistent liver injury and chronic inflammation being the main cause of HCC (27). According to the BCLC staging system, radical treatments (surgical resection, liver transplantation) are recommended as the first line of treatment for HCC stage 0-A. However, for stage B HCC in BCLC, TACE is the most commonly recommended treatment (28). Another studies have shown that overall survival (OS) is superior to other non-radical treatments in BCLC stage B patients with tumors confined to the same liver segment or the ipsilateral hemihepatic region, adequate residual liver volume and adequate tumor-free resection margins (29–31). However, patients with BCLC stage 0-B had high rates of postoperative recurrence despite radical resection due to high risk factors for recurrence (15–17). The presence of high-risk recurrence factors suggests an increased likelihood of residual tumor in the liver. MVI refers to the microscopic presence of HCC cells within the portal vein or vascular lumen, lined by endothelial cells, adjacent to the primary tumor (32). Acting as a “seed” for intrahepatic micrometastases through the hepatic artery or portal venous system, MVI is an independent risk factor for early recurrence in solitary HCC without macroscopic vascular invasion (33). Notably, HCC patients with MVI have a recurrence rate exceeding 20%, and a 5-year overall survival rate of only 24% (34). In addition, large tumors (≥5 cm) or multifocal lesions may harbor undetectable microscopic residual disease before or during surgery, or may lead to intrahepatic dissemination via tumor cell shedding during resection (35, 36). Moreover, poorly differentiated tumors are typically more aggressive and may have already established intrahepatic micrometastases prior to resection (37). Therefore, how to reduce the rate of recurrence after radical resection is still an issue worthy of further research.

Several studies have shown that HAIC has excellent efficacy in postoperative adjuvant therapy for HCC patients with high risk of recurrence, significantly reducing the recurrence rates and prolonging the RFS and OS of patients (19, 38, 39), mainly due to the mechanism of action of chemotherapeutic agents and the unique advantages of HAIC. The chemotherapy regimens used for HAIC in this study were all oxaliplatin combined with 5-fluorouracil (5-Fu). In addition to inhibiting DNA replication and transcription to damage tumour cell DNA, oxaliplatin enhances tumor microenvironment signalling, induces tumor-specific responses and increases tumour cell sensitivity to killer lymphocytes, resulting in anti-tumor activity (40, 41). 5-Fu can be converted to a number of active metabolites (e.g., fluorouridine monophosphate, fluorouridine diphosphate, fluorodeoxyuridine triphosphate, etc.) which interfere with DNA and RNA synthesis and also inhibit the synthesis of deoxythymidine monophosphate (42, 43). HAIC delivers oxaliplatin and 5-Fu directly into the hepatic artery of the liver at high local drug concentrations, improving the efficacy of the drugs against HCC. Meanwhile, due to the first-pass effect of hepatic clearance, the drugs are usually metabolised in the liver, resulting in lower drug concentrations in the peripheral blood and fewer side effects (44). The efficacy of PA-HAIC was also further validated in our study. In our study, PA-HAIC significantly prolonged the RFS and reduced the recurrence rate of patients compared to LR, both in the entire cohort and in the PSM cohort. However, during treatment, chemotherapeutic drugs, while exerting anti-tumor effects, also have certain effects on the tumour microenvironment, promoting secretion of inflammatory factors and suppressing lymphocyte immune function, leading to reduced sensitivity of residual tumour cells to chemotherapeutic drugs (45, 46). This was validated in this study. The effect of the number of PA-HAIC treatments on HCC patient was explored in subgroup analyses and it was ultimately found that the RFS of patients treated with two PA-HAIC treatments was comparable to that of patients who received three or more PA-HAIC treatments and better than that of those who received one PA-HAIC treatment.

MVI disseminate mainly via portal venous branches and spread along as well as against the direction of the portal venous flow, which is thought to have great impact on HCC recurrence (47). It correlates with histological grade, tumor diameter and number of nodules, with a 30%–60% chance of being present in 2–5 cm nodules and up to 60%–90% in >5 cm nodules, and it promotes residual tumor growth and intrahepatic metastasis, leading to early recurrence after radical liver resection (48–51). Numerous studies have demonstrated the high efficacy of postoperative adjuvant therapy in HCC patients with MVI (52–54). A phase III randomized controlled clinical trial investigating the efficacy of PA-HAIC in HCC patients with MVI after radical resection found that mRFS was 20.30 months (95% CI, 10.40–30.30 months) and 10.00 months (95% CI, 6.80–13.20 months) between the PA-HAIC and LR groups, respectively (*p* = 0. 001). The 1-year, 3-year, and 5-year RFS rates were 62.20% (95% CI, 54.20–71.30%), 46.80% (95% CI, 38.00–57.60%), 41.10% (95% CI, 31.80–53.00%) in the PA-HAIC group and 47.20% (95% CI, 39.20–56.70%), 30.10% (95% CI, 22.10–41.00%), 22.60% (95% CI, 14.80–34.50%) in the LR group, with similar results to our study (18).

In addition, the finding that the RFS of patients after radical resection progressively decreased with increasing number and diameter of tumors was further validated in our study (55). And the study further found that among the patients who received PA-HAIC, except for the simple with MVI, the RFS of the patients with MVI + tumor diameter ≥5 cm, MVI + multiple tumor and MVI + tumor diameter ≥5cm + multiple tumor were significantly better than that of the LR group, which proved the good effect of PA-HAIC. Our team speculates that the possible reasons for the non-significant difference in RFS between patients in the PA-HAIC group and the LR group with MVI alone are, first, the proportion of patients in the PA-HAIC group who relapsed by the end of follow-up was lower than that of patients in the LR group; and second, the patients who with MVI alone had a relatively low risk of relapse and longer RFS. Therefore, further extension of the follow-up period is needed in subsequent studies. Among the patients who received PA-HAIC, there was no significant difference in the RFS of MVI + tumor diameter ≥5 cm, MVI + multiple tumor and MVI + tumor diameter ≥5 cm + multiple tumor, which also proved the great efficacy of PA-HAIC.

Age also plays a role in tumor recurrence, with younger patients often experiencing a higher risk of recurrence compared to older patients. Two studies involving HCC patients from multiple centers across China ultimately demonstrated that younger patients, compared to older ones, exhibit greater tumor invasiveness and metastatic potential, leading to higher postoperative recurrence rates and tumor-specific mortality (56, 57). Furthermore, multivariable Cox regression analysis in our study revealed that with increasing age, RFS progressively improved.

This study has several limitations. First, this study was a single-centre retrospective study with a small number of patients enrolled, which led to some bias in the analysis, especially in the subgroup analysis, and further validation of the relevant findings is still needed for multicentre and large-sample studies. Second, due to the relatively short follow-up period, especially the short follow-up period of PA-HAIC, which made it impossible to obtain the OS of the patients, and the relatively single outcome index, we will increase the number of patients and extend the follow-up period in future studies. Third, as some of the pathological findings were not stratified for MVI, this study could not further analyze the prognosis of patients according to MVI stratification.

In conclusion, this study suggests that PA-HAIC is a protective factor for RFS in HCC patients with MVI. Patients aged ≤55 years with MVI + tumor diameter ≥5 cm, MVI + multiple tumors, MVI + tumor diameter ≥5 cm + multiple tumors should receive at least two PA-HAIC treatments.

## Data Availability

The original contributions presented in the study are included in the article/Supplementary Material, further inquiries can be directed to the corresponding authors.

## References

[1] Kocarnik JM Compton K Dean FE Fu W Gaw BL Harvey JD Cancer incidence, mortality, years of life lost, years lived with disability, and disability-adjusted life years for 29 cancer groups from 2010 to 2019: a systematic analysis for the global burden of disease study 2019. JAMA Oncol. (2022) 8(3):420–44. 10.1001/jamaoncol.2021.6987 34967848 PMC8719276

[2] Bray F Ferlay J Soerjomataram I Siegel RL Torre LA Jemal A. Global cancer statistics 2018: GLOBOCAN estimates of incidence and mortality worldwide for 36 cancers in 185 countries. CA Cancer J Clin. (2018) 68(6):394–424. 10.3322/caac.21492 30207593

[3] Zheng S Chan SW Liu F Liu J Chow PKH Toh HC Hepatocellular carcinoma: current drug therapeutic status, advances and challenges. Cancers (Basel). (2024) 16:1582. 10.3390/cancers16081582 38672664 PMC11048862

[4] Maucort-Boulch D de Martel C Franceschi S Plummer M. Fraction and incidence of liver cancer attributable to hepatitis B and C viruses worldwide. Int J Cancer. (2018) 142:2471–7. 10.1002/ijc.31280 29388206

[5] Liu Y Wu F. Global burden of aflatoxin-induced hepatocellular carcinoma: a risk assessment. Environ Health Perspect. (2010) 118:818–24. 10.1289/ehp.0901388 20172840 PMC2898859

[6] Ioannou GN Green P Kerr KF Berry K. Models estimating risk of hepatocellular carcinoma in patients with alcohol or NAFLD-related cirrhosis for risk stratification. J Hepatol. (2019) 71:523–33. 10.1016/j.jhep.2019.05.008 31145929 PMC6702126

[7] Yang JD Hainaut P Gores GJ Amadou A Plymoth A Roberts LR. A global view of hepatocellular carcinoma: trends, risk, prevention and management. Nat Rev Gastroenterol Hepatol. (2019) 16(10):589–604. 10.1038/s41575-019-0186-y 31439937 PMC6813818

[8] Benson AB D’Angelica MI Abbott DE Anaya DA Anders R Are C Hepatobiliary cancers, version 2.2021, NCCN clinical practice guidelines in oncology. J Natl Compr Canc Netw. (2021) 19(5):541–65. 10.6004/jnccn.2021.0022 34030131

[9] Sun Y Zhang W Bi X Yang Z Tang Y Jiang L Systemic therapy for hepatocellular carcinoma: Chinese consensus-based interdisciplinary expert statements. Liver Cancer. (2022) 11(3):192–208. 10.1159/000521596 35949289 PMC9218612

[10] EASL clinical practice guidelines: management of hepatocellular carcinoma. J Hepatol. (2018) 69:182–236. 10.1016/j.jhep.2018.03.019 29628281

[11] Bruix J Gores GJ Mazzaferro V. Hepatocellular carcinoma: clinical frontiers and perspectives. Gut. (2014) 63:844–55. 10.1136/gutjnl-2013-306627 24531850 PMC4337888

[12] Kim J Kang W Sinn DH Gwak GY Paik YH Choi MS Substantial risk of recurrence even after 5 recurrence-free years in early-stage hepatocellular carcinoma patients. Clin Mol Hepatol. (2020) 26(4):516–28. 10.3350/cmh.2020.0016 32911589 PMC7641570

[13] Tsilimigras DI Bagante F Moris D Hyer JM Sahara K Paredes AZ Recurrence patterns and outcomes after resection of hepatocellular carcinoma within and beyond the Barcelona clinic liver cancer criteria. Ann Surg Oncol. (2020) 27(7):2321–31. 10.1245/s10434-020-08452-3 32285278

[14] Zhu XD Li KS Sun HC. Adjuvant therapies after curative treatments for hepatocellular carcinoma: current status and prospects. Genes Dis. (2020) 7:359–69. 10.1016/j.gendis.2020.02.002 32884990 PMC7452398

[15] Guo B Chen Q Liu Z Chen X Zhu P. Adjuvant therapy following curative treatments for hepatocellular carcinoma: current dilemmas and prospects. Front Oncol. (2023) 13:1098958. 10.3389/fonc.2023.1098958 37139151 PMC10149944

[16] Wakayama K Kamiyama T Yokoo H Orimo T Shimada S Einama T Huge hepatocellular carcinoma greater than 10 cm in diameter worsens prognosis by causing distant recurrence after curative resection. J Surg Oncol. (2017) 115(3):324–9. 10.1002/jso.24501 28192617

[17] Nitta H Allard MA Sebagh M Ciacio O Pittau G Vibert E Prognostic value and prediction of extratumoral microvascular invasion for hepatocellular carcinoma. Ann Surg Oncol. (2019) 26(8):2568–76. 10.1245/s10434-019-07365-0 31054040

[18] Li SH Mei J Cheng Y Li Q Wang QX Fang CK Postoperative adjuvant hepatic arterial infusion chemotherapy with FOLFOX in hepatocellular carcinoma with microvascular invasion: a multicenter, phase III, randomized study. J Clin Oncol. (2023) 41(10):1898–908. 10.1200/jco.22.01142 36525610 PMC10082249

[19] Hu L Zheng Y Lin J Shi X Wang A. Does adjuvant hepatic artery infusion chemotherapy improve patient outcomes for hepatocellular carcinoma following liver resection? A meta-analysis. World J Surg Oncol. (2023) 21:121. 10.1186/s12957-023-03000-1 37013589 PMC10069128

[20] Feng X Feng GY Tao J Ao YP Wu XH Qi SG Comparison of different adjuvant therapy regimen efficacies in patients with high risk of recurrence after radical resection of hepatocellular carcinoma. J Cancer Res Clin Oncol. (2023) 149(12):10505–18. 10.1007/s00432-023-04874-0 37284841 PMC11797173

[21] Wang QB Li J Zhang ZJ Li YK Liang YB Chen XM The effectiveness and safety of therapies for hepatocellular carcinoma with tumor thrombus in the hepatic vein, inferior vena cave and/or right atrium: a systematic review and single-arm meta-analysis. Expert Rev Anticancer Ther. (2025) 25(5):561–70. 10.1080/14737140.2025.2489651 40181594

[22] Liver cancer committee of Chinese anti-cancer association LcgoCaoh, Pathology committee of Chinese anti-cancer association. Evidence-based practice guidelines for standardized pathological diagnosis of primary liver cancer in China: 2015. Zhonghua Gan Zang Bing Za Zhi. (2015) 23(5):321–7. 10.3969/j.issn.1001-5256.2015.06.004 26427066 PMC12770223

[23] Xu XF Diao YK Zeng YY Li C Li FW Sun LY Association of severity in the grading of microvascular invasion with long-term oncological prognosis after liver resection for early-stage hepatocellular carcinoma: a multicenter retrospective cohort study from a hepatitis B virus-endemic area. Int J Surg. (2023) 109(4):841–9. 10.1097/js9.0000000000000325 36974673 PMC10389398

[24] Zhou XD Tang ZY Yang BH Lin ZY Ma ZC Ye SL Experience of 1,000 patients who underwent hepatectomy for small hepatocellular carcinoma. Cancer. (2001) 91(8):1479–86. 10.1002/1097-0142(20010415)91:8<1479::aid-cncr1155>3.0.co;2-0 11301395

[25] Makuuchi M Hasegawa H Yamazaki S. Ultrasonically guided subsegmentectomy. Surg Gynecol Obstet. (1985) 161:346–50. 2996162

[26] He MK Le Y Li QJ Yu ZS Li SH Wei W Hepatic artery infusion chemotherapy using mFOLFOX versus transarterial chemoembolization for massive unresectable hepatocellular carcinoma: a prospective non-randomized study. Chin J Cancer. (2017) 36(1):83. 10.1186/s40880-017-0251-2 29061175 PMC5654007

[27] Yu LX Ling Y Wang HY. Role of nonresolving inflammation in hepatocellular carcinoma development and progression. NPJ Precis Oncol. (2018) 2:6. 10.1038/s41698-018-0048-z 29872724 PMC5871907

[28] Bruix J Reig M Sherman M. Evidence-based diagnosis, staging, and treatment of patients with hepatocellular carcinoma. Gastroenterology. (2016) 150:835–53. 10.1053/j.gastro.2015.12.041 26795574

[29] Huang CT Chu YL Su TH Huang SC Tseng TC Hsu SJ Optimizing survival benefit by surgical resection by the seven-eleven criteria in Barcelona clinic liver cancer stage A/B hepatocellular carcinoma beyond the Milan criteria. Liver Cancer. (2023) 12(6):539–49. 10.1159/000529143 38476293 PMC10928811

[30] Kim JY Sinn DH Gwak GY Choi GS Saleh AM Joh JW Transarterial chemoembolization versus resection for intermediate-stage (BCLC B) hepatocellular carcinoma. Clin Mol Hepatol. (2016) 22(2):250–8. 10.3350/cmh.2016.0015 27377909 PMC4946408

[31] Torzilli G Belghiti J Kokudo N Takayama T Capussotti L Nuzzo G A snapshot of the effective indications and results of surgery for hepatocellular carcinoma in tertiary referral centers: is it adherent to the EASL/AASLD recommendations?: an observational study of the HCC east-west study group. Ann Surg. (2013) 257(5):929–37. 10.1097/SLA.0b013e31828329b8 23426336

[32] Roayaie S Blume IN Thung SN Guido M Fiel MI Hiotis S A system of classifying microvascular invasion to predict outcome after resection in patients with hepatocellular carcinoma. Gastroenterology. (2009) 137(3):850–5. 10.1053/j.gastro.2009.06.003 19524573 PMC2739450

[33] Hirokawa F Hayashi M Asakuma M Shimizu T Inoue Y Uchiyama K. Risk factors and patterns of early recurrence after curative hepatectomy for hepatocellular carcinoma. Surg Oncol. (2016) 25(1):24–9. 10.1016/j.suronc.2015.12.002 26979637

[34] Yamashita Y Tsuijita E Takeishi K Fujiwara M Kira S Mori M Predictors for microinvasion of small hepatocellular carcinoma ≤2 cm. Ann Surg Oncol. (2012) 19(6):2027–34. 10.1245/s10434-011-2195-0 22203184

[35] Liu S Li H Guo L Zhang B Zhou B Zhang W Tumor size affects efficacy of adjuvant transarterial chemoembolization in patients with hepatocellular carcinoma and microvascular invasion. Oncologist. (2019) 24(4):513–20. 10.1634/theoncologist.2018-0305 30552155 PMC6459238

[36] Portolani N Coniglio A Ghidoni S Giovanelli M Benetti A Tiberio GA Early and late recurrence after liver resection for hepatocellular carcinoma: prognostic and therapeutic implications. Ann Surg. (2006) 243(2):229–35. 10.1097/01.sla.0000197706.21803.a1 16432356 PMC1448919

[37] Kenmochi K Sugihara S Kojiro M. Relationship of histologic grade of hepatocellular carcinoma (HCC) to tumor size, and demonstration of tumor cells of multiple different grades in single small HCC. Liver. (1987) 7:18–26. 10.1111/j.1600-0676.1987.tb00310.x 3033422

[38] Li S Mei J Wang Q Guo Z Lu L Ling Y Postoperative adjuvant transarterial infusion chemotherapy with FOLFOX could improve outcomes of hepatocellular carcinoma patients with microvascular invasion: a preliminary report of a phase III, randomized controlled clinical trial. Ann Surg Oncol. (2020) 27(13):5183–90. 10.1245/s10434-020-08601-8 32418078

[39] Ke Q Wang L Wu W Huang X Li L Liu J Meta-analysis of postoperative adjuvant hepatic artery infusion chemotherapy versus surgical resection alone for hepatocellular carcinoma. Front Oncol. (2021) 11:720079. 10.3389/fonc.2021.720079 35004268 PMC8727591

[40] Hato SV Khong A de Vries IJ Lesterhuis WJ. Molecular pathways: the immunogenic effects of platinum-based chemotherapeutics. Clin Cancer Res. (2014) 20:2831–7. 10.1158/1078-0432.Ccr-13-3141 24879823

[41] Gatti L Cassinelli G Zaffaroni N Lanzi C Perego P. New mechanisms for old drugs: insights into DNA-unrelated effects of platinum compounds and drug resistance determinants. Drug Resist Updat. (2015) 20:1–11. 10.1016/j.drup.2015.04.001 26003720

[42] Sethy C Kundu CN. 5-Fluorouracil (5-FU) resistance and the new strategy to enhance the sensitivity against cancer: implication of DNA repair inhibition. Biomed Pharmacother. (2021) 137:111285. 10.1016/j.biopha.2021.111285 33485118

[43] Zhang N Yin Y Xu SJ Chen WS. 5-Fluorouracil: mechanisms of resistance and reversal strategies. Molecules. (2008) 13:1551–69. 10.3390/molecules13081551 18794772 PMC6244944

[44] Song MJ. Hepatic artery infusion chemotherapy for advanced hepatocellular carcinoma. World J Gastroenterol. (2015) 21:3843–9. 10.3748/wjg.v21.i13.3843 25852268 PMC4385530

[45] Zhang F Hu K Liu W Quan B Li M Lu S Oxaliplatin-resistant hepatocellular carcinoma drives immune evasion through PD-L1 up-regulation and PMN-singular recruitment. Cell Mol Gastroenterol Hepatol. (2023) 15(3):573–91. 10.1016/j.jcmgh.2022.12.002 36513250 PMC9868681

[46] Xu M Zhao Z Song J Lan X Lu S Chen M Interactions between interleukin-6 and myeloid-derived suppressor cells drive the chemoresistant phenotype of hepatocellular cancer. Exp Cell Res. (2017) 351(2):142–9. 10.1016/j.yexcr.2017.01.008 28109867

[47] Lim KC Chow PK Allen JC Chia GS Lim M Cheow PC Microvascular invasion is a better predictor of tumor recurrence and overall survival following surgical resection for hepatocellular carcinoma compared to the Milan criteria. Ann Surg. (2011) 254(1):108–13. 10.1097/SLA.0b013e31821ad884 21527845

[48] Llovet JM Schwartz M Mazzaferro V. Resection and liver transplantation for hepatocellular carcinoma. Semin Liver Dis. (2005) 25:181–200. 10.1055/s-2005-871198 15918147

[49] Erstad DJ Tanabe KK. Prognostic and therapeutic implications of microvascular invasion in hepatocellular carcinoma. Ann Surg Oncol. (2019) 26:1474–93. 10.1245/s10434-019-07227-9 30788629

[50] Wang XH Liu QB Xiang CL Mao XH Yang B Li Q Multi-institutional validation of novel models for predicting the prognosis of patients with huge hepatocellular carcinoma. Int J Cancer. (2021) 149(1):127–38. 10.1002/ijc.33516 33586134

[51] Kudo M Kitano M Sakurai T Nishida N. General rules for the clinical and pathological study of primary liver cancer, nationwide follow-up survey and clinical practice guidelines: the outstanding achievements of the liver cancer study group of Japan. Dig Dis. (2015) 33:765–70. 10.1159/000439101 26488173

[52] Xiang C Shen X Zeng X Zhang Y Ma Z Zhang G Effect of transarterial chemoembolization as postoperative adjuvant therapy for intermediate-stage hepatocellular carcinoma with microvascular invasion: a multicenter cohort study. Int J Surg. (2024) 110(1):315–23. 10.1097/js9.0000000000000805 37812183 PMC10793739

[53] Yang J Liang H Hu K Xiong Z Cao M Zhong Z The effects of several postoperative adjuvant therapies for hepatocellular carcinoma patients with microvascular invasion after curative resection: a systematic review and meta-analysis. Cancer Cell Int. (2021) 21(1):92. 10.1186/s12935-021-01790-6 33549093 PMC7868028

[54] Bai S Hu L Liu J Sun M Sun Y Xue F. Prognostic nomograms combined adjuvant lenvatinib for hepatitis B virus-related hepatocellular carcinoma with microvascular invasion after radical resection. Front Oncol. (2022) 12:919824. 10.3389/fonc.2022.919824 35898866 PMC9309730

[55] Qi YP Zhong JH Liang ZY Zhang J Chen B Chen CZ Adjuvant transarterial chemoembolization for patients with hepatocellular carcinoma involving microvascular invasion. Am J Surg. (2019) 217(4):739–44. 10.1016/j.amjsurg.2018.07.054 30103903

[56] Diao YK Liu JW Wu H Wang MD Fan XP Chen TH Long-term oncologic outcomes of liver resection for hepatocellular carcinoma in adolescents and young adults: a multicenter study from a hepatitis B virus-endemic area. Am J Surg. (2021) 222(4):751–8. 10.1016/j.amjsurg.2021.03.009 33741185

[57] Pu JL Chen Z Yao LQ Feng JY Diao YK Guan MC Long-term oncological prognosis after curative-intent liver resection for hepatocellular carcinoma in the young versus the elderly: multicentre propensity score-matching study. BJS Open. (2022) 6:zrab145. 10.1093/bjsopen/zrab145 35086147 PMC8794648

