# Supplementary material for: Efficacy of postoperative adjuvant hepatic artery infusion chemotherapy for hepatocellular carcinoma in microvascular invasion: a propensity-matched score
Source: Front Surg. 2025 Oct 3;12:1619772. doi: 10.3389/fsurg.2025.1619772 (PMC12531241; doi:10.3389/fsurg.2025.1619772)

Supplementary Material

Efficacy of Postoperative Adjuvant Hepatic Artery Infusion Chemotherapy for Hepatocellular Carcinoma in Microvascular Invasion: A Propensity-Matched Score

**Xu Feng^1^, Xin-Hua Wu^1^, Kai Chen^1^, Yu-Pei Ao^2^, Zheng-Rong Shi^1^,** Yi-Xuan Gong^1^**^*^**

^1^Department of Hepatobiliary Surgery, The First Affiliated Hospital of Chongqing Medical University, (China), Chongqing

^2^Health Screening Centre, Chongqing Western Hospital, (China), Chongqing

^3^Department of Hepatobiliary Surgery, The Sixth People’s Hospital of Deyang, Sichuan, China

Correspondence authors: Yi-Xuan Gong, 1023669232@qq.com

Supplementary Table 1 Univariate and multivariate survival analysis of RFS

| Characteristics | | the entire cohort | | the PSM cohort | |
| --- | --- | --- | --- | --- | --- |
|  |  | HR (95% CI) | p | HR (95% CI) | p |
| Treatment | LR | Reference |  | Reference |  |
|  | PA-HAIC | 0.411 (0.278, 0.608) | ＜0.001 | 0.489 (0.319, 0.749) | ＜0.001 |
| Age | | 0.991 (0.974, 1.008) | 0.296 |  | 0.970 (0.948, 0.992) |
| AFP | | 1.00 (1.00,1.00) | 0.268 |  | 1.000 (1.000, 1.000) |
| Tumor diameter | | 1.093 (1.023, 1.168) | 0.008 | 2.246 (1.491, 3.383) | 1.096 (1.007, 1.193) |
| Sex | Female | Reference |  | Reference |  |
|  | Male | 1.125 (0.653, 1.938) | 0.671 | 1.016 (0.551, 1.872) | 0.959 |
| HBV | Negative | Reference |  | Reference |  |
|  | Positive | 0.811 (0.461, 1.425) | 0.466 | 0.845 (0.407, 1.755) | 0.652 |
| Liver Cirrhosis | Negative | Reference |  | Reference |  |
|  | Positive | 0.802 (0.549, 1.171) | 0.252 | 0.719 (0.466, 1.109) | 0.136 |
| Tumor number | Single | Reference |  | Reference |  |
|  | Multiple | 1.739 (1.179, 2.564) | 0.005 | 1.662 (1.042, 2.653) | 0.033 |
| BCLC grade | 0+A1 | Reference |  | Reference |  |
|  | A2 | 1.710 (0.944, 3.098) | 0.770 | 1.668 (0/874, 3.184) | 0.121 |
|  | B | 1.752 (1.129, 2.720) | 0.012 | 1.658 (0.993, 2.948) | 0.085 |
| Differentiation | High | Reference |  | Reference |  |
|  | Median | 1.718 (0.918, 3.315) | 0.091 | 1.789 (0.861, 3.716) | 0.119 |
|  | Low | 1.817 (0.787, 4.199) | 0.162 | 3.146 (1.135, 8.719) | 0.028 |
| Resection pattern | Anatomic | Reference |  | Reference |  |
|  | Nonanatomic | 0.956 (0.649, 1.410) | 0.821 | 0.971 (0.624, 1.510) | 0.895 |
| Child-Pugh grade | A | Reference |  | Reference |  |
|  | B | 1.561 (0.721, 3.383) | 0.259 | 1.881 (0.753, 4.698) | 0.176 |
| Hemoglobin | | 1.004 (0.996, 1.013) | 0.313 |  | 1.006 (0.997, 1.016) |
| NLR | | 1.120 (1.048, 1.197) | ＜0.001 | 1.052 (0.896, 1.237) | 1.162 (1.066, 1.267) |
| PLR | | 1.002 (0.999, 1.005) | 0.172 |  | 1.000 (0.996, 1.004) |
| SII | | 1.000 (1.000, 1.001) | 0.012 | 1.000 (0.999, 1.001) | 1.000 (1.000, 1.001) |
| Total protein | | 0.995 (0.970, 1.020) | 0.676 |  | 0.982 (0.954, 1.011) |
| Albumin | | 0.998 (0.963, 1.034) | 0.906 |  | 0.991 （0.949, 1.034） |
| Total bilirubin | | 1.017 (0.996, 1.038) | 0.108 |  | 1.020 (0.998, 1.042) |
| ALT | | 0.978 (0.992,1.002) | 0.299 |  | 1.000 (0.994, 1.006) |
| AST | | 1.000 (0.997, 1.004) | 0.872 |  | 1.004 (0.996, 1.011) |
| PT | | 1.118 (0.902, 1.384) | 0.308 |  | 1.289 (1.003, 1.658) |
| Hemorrhage | | 1.000 (1.000,1.000) | 0.645 |  | 1.000 (1.000, 1.000) |
| Operating time | | 1.000 (0.998, 1.002) | 0.806 |  | 1.001 (0.999. 1.003) |
| Blood transfusion | No | Reference |  | Reference |  |
|  | Yes | 0.889 (0.463, 1.709) | 0.723 | 0.780 (0.360, 1.693) | 0.530 |

PSM- propensity score-matching, LR- liver resection, PA-HAIC- postoperative adjuvant hepatic arterial infusion chemotherapy, AFP - Alpha-fetoprotein, HBV- Hepatitis B virus, BCLC- Barcelona clinic liver cancer, NLR=neutrophil/lymphocyte, PLR=platelet/lymphocyte, SII=neutrophil*platelet/lymphocyte, SIRI=neutrophil*monocyte/lymphocyte; ALT -Alanine aminotransferase, AST -Aspartate aminotransferase, PT- Prothrombin Time

Supplementary Figure 1 Kaplan-Meier analysis of recurrence-free survival in HCC patients with different risks of recurrence (the entire cohort)


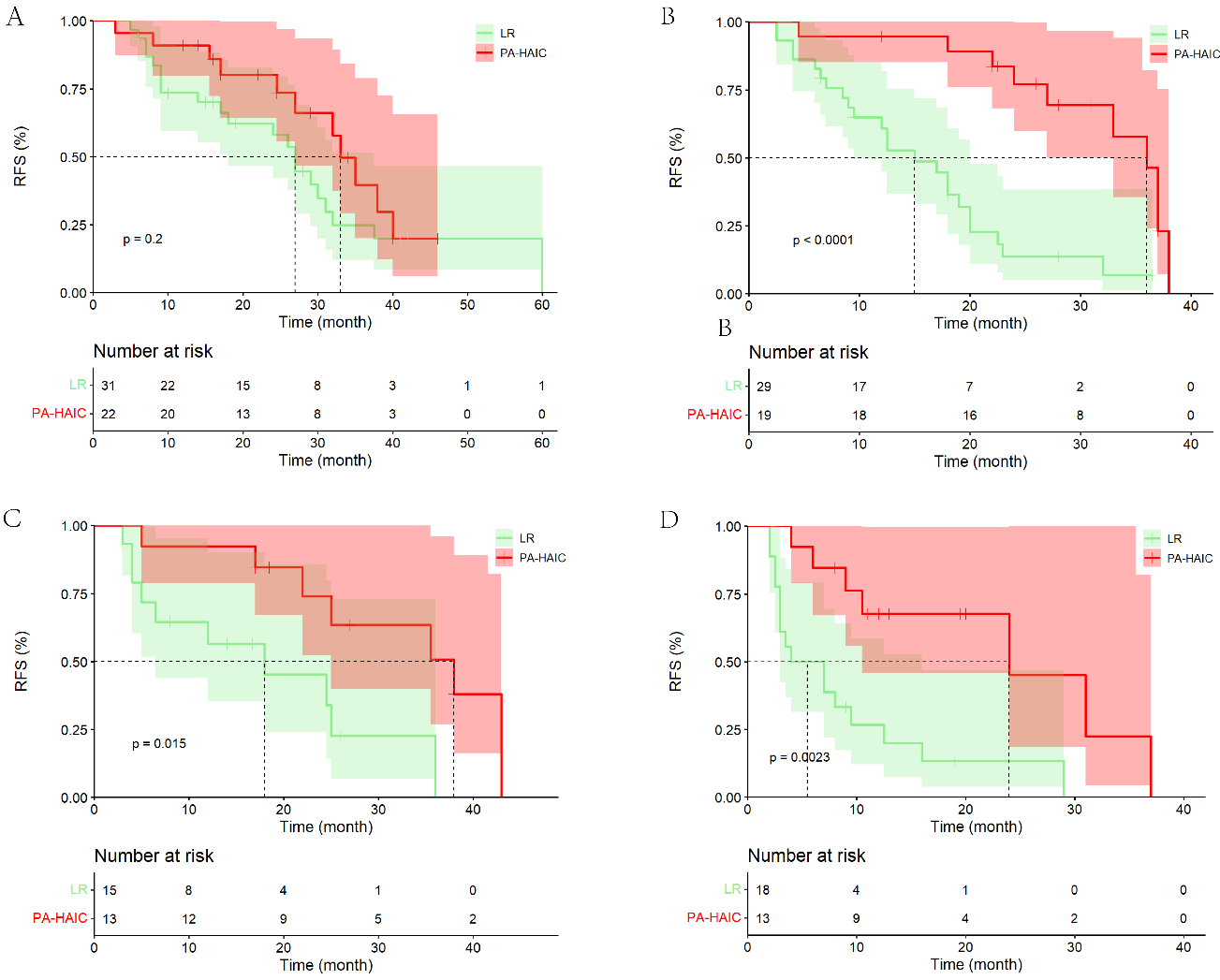


A—with MVI; B—with MVI + tumor diameter ≥ 5cm; C—with MVI + multiple tumor, D—with MVI + tumor diameter ≥ 5cm and multiple tumor

Supplementary Table 2.A the recurrence-free survival of patients with different risks of recurrence (the entire cohort)

|  | RFS | p | | | |
| --- | --- | --- | --- | --- | --- |
|  |  | MVI | MVID | MVIN | MVID+N |
| MVI | 27.00 (22.66，31.34) |  | 0.012 | 0.043 | ＜0.001 |
| MVID | 15.00 (9.13, 20.88) | 0.012 |  | 0.559 | 0.014 |
| MVIN | 22.00 (22.66, 33.17) | 0.043 | 0.559 |  | 0.048 |
| MVID+N | 4.00 (0, 8.85) | ＜0.001 | 0.014 | 0.048 |  |

MVI—with MVI alone, MVID—with MVI + tumor diameter ≥ 5cm; MVIN—with MVI +multiple tumor, MVID+N—with MVI + tumor diameter ≥ 5cm and multiple tumor

Supplementary Table 2.B the recurrence-free survival of patients with different risks of recurrence (the PSM cohort)

|  | RFS | p | | | |
| --- | --- | --- | --- | --- | --- |
|  |  | MVI | MVID | MVIN | MVID+N |
| MVI | 27.00 (21.51，32.49) |  | 0.008 | 0.038 | ＜0.001 |
| MVID | 18.00 (15.49, 20.51) | 0.008 |  | 0.692 | ＜0.001 |
| MVIN | 15.00 (0, 32.33) | 0.038 | 0.692 |  | 0.010 |
| MVID+N | 3.00 (1.80, 4.20) | ＜0.001 | ＜0.001 | 0.010 |  |

MVI—with MVI alone, MVID—with MVI + tumor diameter ≥ 5cm; MVIN—with MVI + multiple tumor, MVID+N—with MVI + tumor diameter ≥ 5cm and multiple tumor

Supplementary Figure 2 Kaplan-Meier analysis of recurrence-free survival in HCC patients with different risk factors in the PA-HAIC group (the entire cohort)


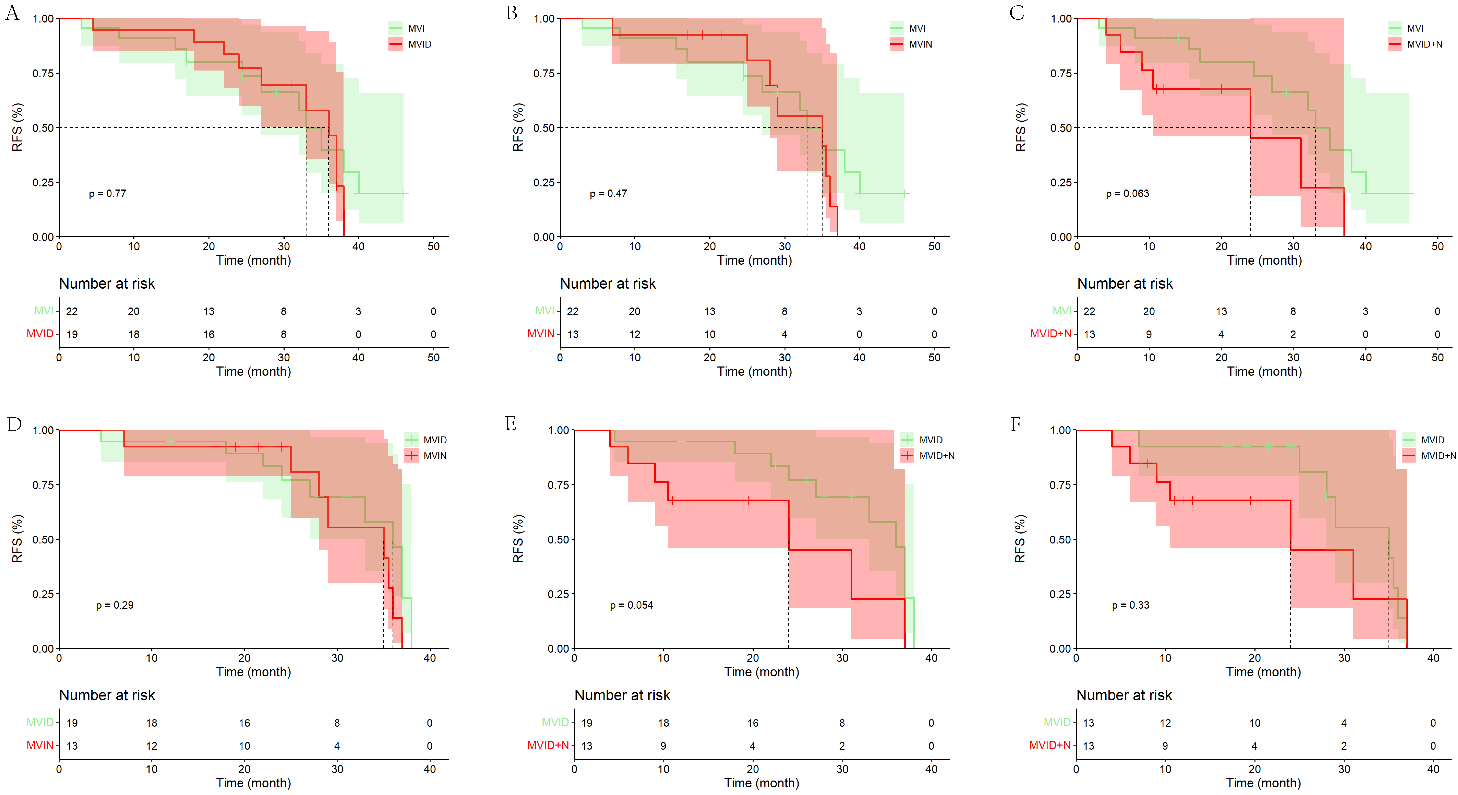

Supplement: Supplementary file 1 [file Supplementaryfile1.docx]
